# Allelic variants in vitamin D receptor gene are associated with adiposity measures in the central-European population

**DOI:** 10.1186/s12881-017-0454-z

**Published:** 2017-08-22

**Authors:** Julie Bienertová-Vašků, Filip Zlámal, Aneta Pohořalá, Ondřej Mikeš, Monika Goldbergová-Pávková, Jan Novák, Zbyněk Šplíchal, Hynek Pikhart

**Affiliations:** 1 Research Centre for Toxic Compounds in the Environment, Faculty of Sciences, Masaryk University, Kamenice A29, Brno, Czech Republic; 2 Department of Pathological Physiology, Faculty of Medicine, Masaryk University, Kamenice A18, Brno, Czech Republic; 3 Research Department of Epidemiology and Public Health, University College London, 1-19 Torrington Place, London, WC1E 6BT UK

**Keywords:** VDR, SNP, Obesity, Anthropometry, Adiposity, Gene, Vitamin D, Polymorphism, Adiposity measure

## Abstract

**Background:**

There is an increasing body of evidence suggesting that vitamin D is involved in ethiopathogenesis of obesity and therefore the aim of the study was to investigate whether 5 selected SNPs in VDR (vitamin D receptor) gene are associated also with anthropometry in the obese and non-obese Central-European population.

**Methods:**

A total of 882 Central European Caucasian individuals of Czech origin were recruited (*n* = 882, 232 M/650 F) and weight, height, BMI, lean body mass, fat mass, body fat, waist and hip circumference, waist–hip ratio (WHR) and skinfold thickness were measured. Univariate and multivariate models were constructed in order to investigate the relationship between anthropometry and VDR polymorphisms.

**Results:**

In the univariate modeling, the CC genotype of FokI SNP was associated with reduced waist circumference (β = −3.48; 95%CI:-7.11;0.15; *p* = 0.060), sum of skin fold thickness (β = −6.53, 95% CI: -12.96;-0.11; *p* = 0.046) as well as total % of body fat (β = −3.14, 95% CI: -5.18;-1.09; *p* = 0.003) compared to TT genotype. The AC genotype of ApaI SNP was associated with reduced waist circumference compared to AA genotype (β = −4.37, 95% CI: -7.54;-1.20; *p* = 0.007). GG genotype of EcoRV SNP was associated with reduced sum of skin fold thickness compared to AA genotype (β = −7.77, 95% CI: -14.34;-1.21; *p* = 0.020). In the multivariate modelling, multiple significant associations of VDR with investigated traits were observed, too.

**Conclusion:**

Our study suggests that genetic variability in the VDR region may be an important factor influencing anthropometric characteristics associated with obesity.

## Background

Obesity is an epidemic worldwide and represents a potential risk factor for many chronic health conditions including cardiometabolic diseases as well as cancer. The increase of obesity prevalence in some geographical regions is, despite an undoubtable genetic contribution, difficult to explain and is attributed mainly to the obesity-associated lifestyle factors, largely manageable by public health interventions. However, the exact proportion of genetic and/or environmental influences in the complex diseases is undoubtedly difficult to estimate.

Apart from its fundamental role in prevention of rickets, low vitamin D status has recently become a major public health problem due to its associations with several chronic metabolic diseases [1]. It is empirically known that obesity is associated with vitamin D status but the underlying pathophysiology has been uncertain and the role of vitamin D receptor variability in obesity is far from being understood. The possible positive associations of vitamin D status with obesity reported in the smaller studies have not been confirmed either in randomized controlled studies testing the effect of vitamin D supplementation on obesity risk [2, 3]. Therefore, the role of vitamin D in obesity prophylaxis and/or treatment remains to be further investigated.

Still, it seems that vitamin D effect on obesity risk is biologically plausible as in vitro experiments in rats have previously shown that administration of extensive doses of vitamin D2 may lead to an increase in energy expenditure due to uncoupling of oxidative phosphorylation in adipose tissues [4]. However, these data cannot be simply extrapolated to humans, as there are numerous differences in obesity pathophysiology between mice and humans.

Generally, vitamin D, that is derived from the diet or from 7-dehydrocholesterol, must be further activated to achieve full activity within the body [4]. The most active known metabolite of vitamin D is calcitriol, i.e. 1,25- dihydroxyvitamin D (1,25(OH)2D), and its effects on genome are mediated through a transcription factor activated by a ligand, vitamin D receptor (VDR) [5]. 1,25(OH)2D functions as a ligand by direct binding to the VDR locus, a key member of the steroid hormone receptor family, which in turn regulates the transcription of the numerous target genes.

The DNA polymorphisms that have been often reported for the VDR gene are: *BsmI* (rs1544410), *ApaI* (rs7975232), TaqI (rs731236), FokI (rs2228570) and a EcoRV site in the promoter region (rs4516035) [6]. In the past years, many studies reported these polymorphisms to be related to bone metabolism, cancer, type 1 diabetes and obesity [7]. However, the association with obesity was reported in some, but not all studies [8]. As vitamin D supplementation is commonly recommended to general population, it is important to know whether the beneficial effects are BMI dependent and more research to the role of vitamin D in body composition is therefore necessary.

In the present study, we investigated the relationship between anthropometric traits including BMI, a widespread measure for monitoring the prevalence of obesity at the population level, and selected genetic variants, namely BsmI, FokI, ApaI, EcoRV (GATA) and TaqI in the VDR gene, in the Central-European obese and lean population.

## Methods

### Subjects

A total of 882 Central European Caucasian individuals of Czech origin were recruited for the study in a mass media campaign targeting the South Moravia region of the Czech Republic (232 men and 650 women; mean BMI 31.9 (SD 7.4) kg/m^2^; median age 50.3 years; age range 18.8–79.7 years). Inclusion and exclusion criteria were reported elsewhere [7, 8]. Briefly, participation in the study was limited to individuals who were 1) not taking cholesterol-lowering medications; 2) were not currently on any lipid-lowering or weight-control diets; 3) were free from possible causes of secondary severe hypercholesterolemia (e.g., hyperthyroidism, pregnancy); and 4) were free from severe chronic illness (e.g., cancer, renal disease, heart failure). However, in contrast to the study by Ma et al., the subjects who worked night shifts were maintained in our study.

The study was conducted according to the guidelines set out in the Declaration of Helsinki and all procedures involving human subjects were approved by the Committee for Ethics of Medical Experiments on Human Subjects, Faculty of Medicine of Masaryk University (Brno, Czech Republic). Written informed consent was obtained from all subjects.

Data on personal or family history of obesity, birth weight, age at onset of obesity and its severity were obtained by a professional interviewer using a semi-structured questionnaire. A positive family history of obesity was estimated as having at least one obese relative with BMI ≥ 30 kg/m2 in the close family (siblings, parents and their siblings, or grandparents). Both the obese cases and the non-obese controls underwent the same examination focused on their anthropometric characteristics; the subjects of the study were also interviewed with respect to their current smoking status and its history, type of work, shift work and their nutritional habits using the 7-day food records on the grounds of which the total energy intake of the individuals was established.

### Anthropometric characteristics

All phenotypic measurements were performed by experienced specialists and included weight, height, BMI, lean body mass, fat mass, body fat, waist and hip circumference, waist–hip ratio (WHR) and skinfold thickness, as described elsewhere [7]. Body composition was assessed by bioelectrical impedance analysis using the InBody 230 bioimpedance analyzer (Biospace CO Ltd., 518–10 Dogok 2-dong, Gangnam-gu, Seoul, Korea) with the subject in a standing position.

### Genotyping

The following SNPs were genotyped: BsmI (rs1544410), ApaI (rs7975232), TaqI (rs731236), FokI (rs2228570) and a EcoRV (GATA) (rs4516035).

The selection of these particular SNPs was based on: (1) its frequency in the European Caucasian population; (2) its presumed functional or regulatory impact on feeding behaviour and its possible functional consequences and (3) a previously described association with obesity. DNA for analyses was extracted from 5 mL of the patients’ saliva collected after 3 h fasting. Genotyping of the polymorphism was performed as described previously using a standard PCR-based methodology with following restriction fragment length polymorphism analysis [9]. Restricted fragments were separated by electrophoresis on 2% agarose gels with ethidium bromide staining. To assess genotyping reliability, we performed double sampling in more than 20% of the samples and found no differences. We always used quality control, and negative controls were used to identify possible false positives.

All reactions were performed using the XP BIOER Cycler (BIOER Technology CO. Ltd., Japan), the overall genotypization success varied between 76.8% (TaqI SNP) and 89.0% (FokI SNP) (with 77.8% for ApaI, 88.8% for BsmI and 88.4% for EcoRV), missing genotypes were due to either consistent PCR dropout or depletion of template DNA.

### Haplotype analysis

The haplotype frequencies were calculated using the method for reconstructing haplotypes from population data according to Stephens et al. [10] Bonferroni correction for multiple hypothesis testing was performed, where appropriate.

### Statistics

Where applicable, it was first determined whether a variable was normally distributed using a battery of normality tests (e.g. Shapiro-Wilk test) and graphical tools; in cases of skewed variables, logarithmic transformation was performed and normal distribution was tested again. For descriptive purposes, mean values are presented using untransformed values. Results are expressed as mean values and standard deviations unless otherwise stated. ANOVA and Kruskal-Wallis test were used for multiple comparisons with subsequent Tukey-Kramer, respectively Benjamini-Hochberg *p*-value correction, where appropriate.

For the purpose of investigation of crude association between genotypes and obesity status, logistic regression was used.

Using the linear regression modelling, weight, height, lean body mass, total body fat, waist and hip circumference, WHR, BMI and total sum of skin folds were modelled as dependent variables, while the genotypes of the investigated SNPs (ApaI, BsmI, EcoRV, Fok-I, Taq-I), gender, smoking status, BMI (squared value) and age as independent variables. Firstly, the univariate models were constructed for the modelled dependent variable in relation to genotypes, separately for each polymorphism.

Consecutively, multivariate models were constructed in relation to variables gender, age, BMI, BMI^2^, smoking status, comorbidity, type of work, shift work, total energy intake and a three-combination of genotype (ApaI-EcoRV-FokI; BsmI-EcoRV-FokI; TaqI-EcoRV-FokI). Type of work was determined as manual/non-manual work and sedentary/non-sedentary work. The multivariate models did not include all the genotypes of the 5 investigated polymorphisms, as the trio Apa-1-Bsm-1-Taq-1 is in strong linkage disequilibrium (LD) and there is a strong statistical dependency between these variables. If two of these SNPs or all the three of them were included into the model, this could significantly bias the confidence intervals in the model due to strong collinearity.

Missing data were imputed using Multiple imputation by chained equations method [11] and the regression models were built using imputed data. The total of 50 imputed datasets was used with the maximum of 20 iterations.

All analyses were performed using R, version 3.1.2 and STATA version 14. Consensual values of *p* < 0.05 were considered statistically significant.

## Results

### Baseline characteristics of the study cohort

The baseline description of the study cohort with respect to demographic and clinical parameters is presented in Table 1. The Hardy–Weinberg disequilibrium was assessed for all sub-cohorts, and none of the SNPs was significantly deviated from it. The relationship between the investigated polymorphisms and obesity status (based on the cut-off value of BMI 30.0 kg/m2) is given in Table 2. Significant differences between the obese and non-obese group were observed for TaqI, where the AA genotype was more frequent in non-obese individual (45.1% vs. 37.1%, *p* = 0.034).

**Table 1:** Basic anthropometric and demographic characteristics of the study subjects

Parameter	Unit	All	Females		Males		*p*-value*
			Non-obese	Obese	Non-obese	Obese	
		882	281	369	90	142	
Height	cm	167.8 ± 8.9	165.6 ± 6.8^abc^	163.6 ± 6.9^ade^	177.8 ± 7.3^bd^	176.9 ± 6.6^ce^	<0.001
Weight	kg	89.8 ± 22.2	68.9 ± 10.3^abc^	98.4 ± 16.9^ade^	82.2 ± 10.5^bdf^	113.8 ± 18.9^cef^	<0.001
BMI	kg/m^2^	31.9 ± 7.4	25.1 ± 3.4^ab^	36.7 ± 5.8^ac^	26.0 ± 2.6^cd^	36.4 ± 5.6^bd^	<0.001
Age	years	48.3 ± 14.2	43.0 ± 14.9^ab^	52.7 ± 12.1^acd^	44.3 ± 15.8^ce^	49.9 ± 12.8^bde^	<0.001
Skinfold thickness (tricipital)	mm	25.1 ± 8.0	20.6 ± 5.6^abc^	30.4 ± 5.9^ade^	16.4 ± 6.2^bdf^	23.6 ± 7.6^cef^	<0.001
Skinfold thickness (bicipital)	mm	18.4 ± 8.0	13.7 ± 5.1^abc^	23.3 ± 7.0^ade^	10.9 ± 5.7^bdf^	17.9 ± 7.3^cef^	<0.001
Skinfold thickness (supraspinal)	mm	22.8 ± 9.1	16.8 ± 5.9^ab^	27.5 ± 8.0^acd^	15.5 ± 6.0^ce^	25.4 ± 9.5^bde^	<0.001
Skinfold thickness (subscapular)	mm	25.7 ± 9.7	18.2 ± 6.3^ab^	30.7 ± 8.3^ac^	18.6 ± 6.6^cd^	29.8 ± 8.3^bd^	<0.001
Skinfold thickness (sum of all)	mm	91.5 ± 30.0	69.1 ± 18.9^abc^	111.6 ± 22.9^ade^	61.4 ± 20.3^bdf^	95.9 ± 25.2^cef^	<0.001
Body fat	%	37.1 ± 10.4	32.2 ± 7.3^abc^	45.8 ± 5.6^ade^	21.4 ± 6.0^bdf^	33.9 ± 6.5^cef^	<0.001
Body water	%	46.4 ± 6.9	49. 6 ± 5.0^ab^	40.8 ± 3.8^acd^	56.4 ± 4.6^bce^	48.4 ± 4.5^de^	<0.001
Waist circumference	cm	101.8 ± 18.6	82.4 ± 10.5^abc^	109.5 ± 12.9^ade^	93.7 ± 9.6^bdf^	119.4 ± 13.7^cef^	<0.001
Hip circumference	cm	113.3 ± 13.9	101.8 ± 7.4^ab^	122.4 ± 12.5^acd^	102.0 ± 5.5^ce^	117.3 ± 10.4^bde^	<0.001
Waist–hip ratio	---	0.90 ± 0.10	0.81 ± 0.08^abc^	0.90 ± 0.08^ade^	0.92 ± 0.07^bdf^	1.02 ± 0.06^cef^	<0.001

For a given variable, each pair of superscript letters (a, b, c, d, e, f) appearing in two diffefent groups (e.g. letter a in non-obese female and obese female group) mean statistically significant difference (*p* < 0.05) in levels of the variable between these groups in post-hoc test, * *p*-value for comparison between obese and non-obese individuals

**Table 2:** VDR polymorphisms and odds of being obese in the study cohort

Polymorphism	Obese	Non-obese	OR (95% CI)*	*p* for linear trend
BsmI	458	325		0.652
CC (%)	178 (38.9)	127 (39.1)	1.00	
CT (%)	213 (46.5)	157 (48.3)	0.97 (0.71;1.32)	
TT (%)	67 (14.6)	41 (12.6)	1.17 (0.74;1.83)	
FokI	460	325		0.055
TT (%)	75 (16.3)	73 (22.5)	1.00	
TC (%)	228 (49.6)	153 (47.1)	0.94 (0.68;1.30)	
CC (%)	157 (34.1)	99 (30.4)	0.65 (0.43;0.98)	
ApaI	407	279		0.195
AA (%)	112 (27.5)	67 (24.0)	1.00	
AC (%)	206 (50.6)	141 (50.5)	0.87 (0.60;1.27)	
CC (%)	89 (21.9)	71 (25.5)	0.75 (0.49;1.16)	
TaqI	402	275		0.034
AA (%)	149 (37.1)	124 (45.1)	1.00	
AG (%)	191 (47.5)	118 (42.9)	1.35 (0.97;1.88)	
GG (%)	62 (15.4)	33 (12.0)	1.56 (0.96;2.54)	
EcoRV	455	325		0.668
AA (%)	131 (28.8)	91 (28.0)	1.00	
AG (%)	235 (51.6)	166 (51.1)	0.98 (0.70;1.37)	
GG (%)	89 (19.6)	68 (20.9)	0.90 (0.60;1.38)	

The given values are outputs of the univariable logistic regression model, ﻿* 95% confidence interval

### Association of investigated VDR polymorphisms with the anthropometric measures

**Fig. 1:**
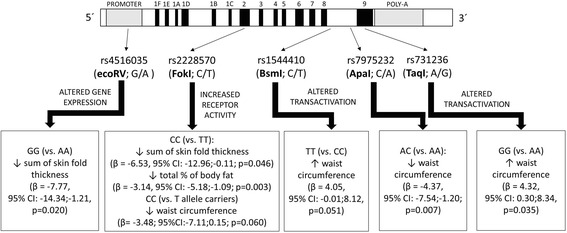
Involvement of vitamin D variability in obesity pathogenesis in relation to the results of the univariate analysis

Briefly, we observed multiple associations of the investigated polymorphisms with anthropometric parameters as shown in Fig. 1.

In the univariable analysis (Table 3), FokI was borderly associated with waist circumference, and CC genotype was associated with reduced waist circumference compared to T allele carriers (β = −3.48, 95% CI: -7.11;0.15, *p* = 0.060). Moreover, CC genotype was also associated with reduction of the sum of skin fold thickness compared to TT genotype (β = −6.53, 95% CI: -12.96;-0.11, *p* = 0.046) as well as reduction of total % of body fat (β = −3.14, 95% CI: -5.18;-1.09, *p* = 0.003). The AC heterozygote genotype of ApaI was associated with reduced waist circumference compared to AA genotype (β = −4.37, 95% CI: -7.54;-1.20, *p* = 0.007). The TT genotype of BsmI was borderly associated with increased waist circumference compared to CC genotype (β = 4.05, 95% CI: -0.01;8.12, *p* = 0.051). GG genotype of EcoRV was associated with reduced sum of skin fold thickness compared to AA genotype (β = −7.77, 95% CI: -14.34;-1.21, *p* = 0.020). The GG genotype of TaqI was associated with increased waist circumference compared to AA genotype (β = 4.32, 95% CI: 0.30;8.34, *p* = 0.035).

**Table 3:** Univariable linear regression coefficients of studied polymorphisms with waist circumference, sum of skinfold thicknesses and total body fat

SNP	Genotypes	Waist circumference [cm]		Sum of skin fold thickness [cm]		Total body fat [%]	
		β estimate (95% CI)	*p*	β estimate (95% CI)	*p*	β estimate (95% CI)	*P*
ApaI	AA	(ref)		(ref)		(ref)	
	AC	**−4.37** **(−7.54;-1.20)**	**0.007**	−2.93 (−8.57;2.71)	0.308	−0.54 (−2.34;1.26)	0.555
	CC	−3.12 (−6.92;0.67)	0.106	−1.85 (−8.62;4.92)	0.591	0.18 (−1.93;2.29)	0.869
BsmI	CC	(ref)		(ref)		(ref)	
	CT	−0.28 (−3.04;2.49)	0.845	−0.54 (−5.59;4.51)	0.834	−0.31 (−1.85;1.23)	0.689
	TT	4.05 (−0.01;8.12)	0.051	−1.72 (−8.35;5.72)	0.714	−1.43 (−3.64;0.78)	0.203
EcoRV	AA	(ref)		(ref)		(ref)	
	AG	0.75 (−2.24;3.73)	0.624	−2.01 (−7.45;3.42)	0.467	−0.88 (−2.57;0.81)	0.305
	GG	−1.32 (−5.05;2.40)	0.486	**−7.77** **(−14.34;-1.21)**	**0.020**	−0.91 (−2.99;1.16)	0.389
FokI	TT	(ref)		(ref)		(ref)	
	TC	−1.94 (−4.79;0.90)	0.181	−4.13 (−9.11;0.86)	0.104	−1.52 (−3.14;0.10)	0.065
	CC	−3.48 (−7.11;0.15)	0.060	**−6.53** **(−12.96;-0.11)**	**0.046**	**−3.14** **(−5.18;-1.09)**	**0.003**
TaqI	AA	(ref)		(ref)		(ref)	
	AG	0.65 (−2.15;3.46)	0.648	3.09 (−2.01;8.18)	0.234	0.33 (−1.24;1.91)	0.678
	GG	**4.32** **(0.30;8.34)**	**0.035**	3.37 (−3.69;10.43)	0.348	−0.18 (−2.47;2.11)	0.876

95%CI 95% confidence interval

The results of the multivariate analysis for the waist circumference, sum of skin fold thickness and total body fat are summarized in Table 4. Briefly, multiple effects of the investigated SNPs on anthropometric characteristics of the study subjects were observed in the multivariate analysis based on genotype three-combinations ApaI-EcoRV-FokI; BsmI-EcoRV-FokI; TaqI-EcoRV-FokI in the model.

**Table 4:** Multivariable models of waist circumference, sum of skin fold thickness and total body fat adjusted for sex, age, BMI, BMI^2^, total energy intake, smoking, type of work, shift work, comorbidities

	SNP	Genotypes	Waist circumference [cm]		Sum of skin fold thickness [cm]		Total body fat [%]	
			β estimate	*p*	β estimate	*p*	β estimate	*p*
Var. 1	ApaI	AA	(ref)		(ref)		(ref)	
		AC	**−1.68** **(−2.88;-0.48)**	**0.006**	−0.45 (−4.19;3.28)	0.811	0.35 (−0.27;0.96)	0.270
		CC	−0.69 (−2.09;0.71)	0.333	0.08 (−4.70;4.86)	0.974	0.56 (−0.16;1.28)	0.130
	EcoRV	AA	(ref)		(ref)		(ref)	
		AG	0.66 (−0.40;1.72)	0.220	−1.12 (−4.76;2.52)	0.545	−0.26 (−0.84;0.33)	0.389
		GG	0.73 (−0.65;2.11)	0.301	**−5.21** **(−9.62;-0.80)**	**0.021**	−0.51 (−1.26;0.23)	0.176
	FokI	TT	(ref)		(ref)		(ref)	
		TC	0.19 (−0.84;1.21)	0.720	−0.66 (−3.81;2.49)	0.680	0.01 (−0.58;0.60)	0.974
		CC	0.01 (−1.28;1.30)	0.987	0.79 (−3.60;5.18)	0.723	−0.21 (−0.95;0.53)	0.584
Var. 2	BsmI	CC	(ref)		(ref)		(ref)	
		CT	−0.50 (−1.50;0.49)	0.319	−0.42 (−3.81;2.96)	0.805	−0.02 (−0.56;0.53)	0.957
		TT	0.70 (−0.82;2.23)	0.364	−1.77 (−6.68;3.15)	0.478	**−1.17** **(−1.95;-0.38)**	**0.004**
	EcoRV	AA	(ref)		(ref)		(ref)	
		AG	0.70 (−0.36;1.76)	0.196	−1.10 (−4.75;2.56)	0.554	−0.25 (−0.84;0.33)	0.392
		GG	0.67 (−0.72;2.07)	0.342	**−5.17** **(−9.59;-0.75)**	**0.022**	−0.47 (−1.21;0.28)	0.218
	FokI	TT	(ref)		(ref)		(ref)	
		TC	0.20 (−0.84;1.23)	0.710	−0.73 (−3.86;2.39)	0.645	−0.04 (−0.63;0.54)	0.883
		CC	−0.03 (−1.32;1.25)	0.959	0.71 (−3.67;5.08)	0.750	−0.26 (−0.99;0.48)	0.492
Var. 3	TaqI	AA	(ref)		(ref)		(ref)	
		AG	**−1.08** **(−2.14;-0.02)**	**0.046**	0.46 (−3.12;4.03)	0.801	−0.30 (−0.86;0.26)	0.292
		GG	−0.35 (−1.86;1.16)	0.649	−1.07 (−5.76;3.61)	0.651	**−0.87** **(−1.67;-0.07)**	**0.033**
	EcoRV	AA	(ref)		(ref)		(ref)	
		AG	0.65 (−0.41;1.71)	0.229	−1.07 (−4.69;2.55)	0.559	−0.25 (−0.84;0.33)	0.398
		GG	0.67 (−0.73;2.07)	0.344	**−5.14** **(−9.54;-0.74)**	**0.022**	−0.51 (−1.25;0.24)	0.180
	FokI	TT	(ref)		(ref)		(ref)	
		TC	0.18 (−0.85;1.21)	0.735	−0.75 (−3.88;2.38)	0.638	−0.03 (−0.61;0.56)	0.931
		CC	0.00 (−1.29;1.29)	0.999	0.67 (−3.74;5.09)	0.763	−0.20 (−0.94;0.53)	0.588

Var: combinations of three genotypes in the given model: Var 1: ApaI-EcoRV-FokI; Var 2: BsmI-EcoRV-FokI; Var 3: TaqI-EcoRV-FokI in the model

The results of haplotype analysis are reported in Table 5, using the obesity status as a dependent variable (Table 5). Briefly, no significant association of any of identified haplotypes with obesity status was observed. Similarly, no significant association of haplotypes was observed when analysing other anthropometric variables (% body fat, skin fold thickness, waist circumference) (data not shown).

**Table 5:** VDR haplotype association with obesity

haplotype	Haplotype frequency in controls	Haplotype frequency in obese cases	OR (95% CI)	*p*-value
CCTGA	0.136	0.136	1	-
CCCAA	0.088	0.052	0.60 (0.29–1.24)	0.166
CTTGA	0.017	0.003	0.24 (0.03–1.77)	0.163
CCTAA	0.116	0.113	0.87 (0.42–1.77)	0.699
ATCAA	0.011	0.009	0.54 (0.15–1.92)	0.341
ACCAA	0.026	0.025	1.13 (0.36–3.52)	0.834
CCCGA	0.127	0.128	1.00 (0.52–1.92)	0.994
ATCAG	0.058	0.056	0.98 (0.45–2.15)	0.960
ACCGA	0.035	0.029	0.70 (0.25–1.96)	0.492
ACTAA	0.046	0.042	0.83 (0.36–1.96)	0.678
ATTAG	0.089	0.096	1.01 (0.55–1.86)	0.977
ATCGG	0.070	0.086	1.18 (0.64–2.18)	0.598
ACTGA	0.024	0.049	1.89 (0.6–5.94)	0.274
ATTGG	0.081	0.102	1.20 (0.59–2.47)	0.613

Benjamini-Hochberg *p*-value correction for multiple hypothesis testing was performed, where appropriate

## Discussion

In this study, we identified multiple associations of selected SNPs in VDR region with anthropometry. More specifically, all the investigated SNPs except for EcoRV (i.e. BsmI, TaqI, FokI, ApaI) were associated with waist circumference. EcoRV and FokI were associated with sum of skin fold thicknesses and FokI was also associated with total % of body fat.

Several studies have observed associations of VDR SNPs with anthropometric traits and yielded contradictory results. In a study by Ferrarezi, BsmI, ApaI and FokI genotypes were significantly associated with height in pubertal, but not prepubertal children, while the homozygous carriers of the minor allele of BsmI were by 0.65 z-scores (approx. equal to 4 cm) higher than the homozygous carriers of the major allele (*p* = 0.0006) [12]. However, these reports contradict a large analysis from 2008 that reports only negligible influence of FokI on height in large samples from Australia (*N* = 3906) and the Netherlands (*N* = 1689) [13]. On the other hand, in a more recent, large Tromsø study on 9471 subjects, significant association of BsmI and ApaI with height was observed [14].

Several studies attempted to link variability in VDR region with obesity and adiposity traits on smaller population samples. In a Saudi cohort, the BsmI minor allele (T allele) was significantly more frequent in obese individuals as well as TaqI (G allele) [15]. In a small Greek cohort, TaqI was significantly associated with obesity, (OR: 2.07, 95% CI: 1.123;3.816, *p* = 0.019), contributing to an elevated BMI of 3 kg/m^2^ per risk allele [16]. Study on 1773 healthy female adults recruited from western New York tested associations of 14 SNPs in VDR with the following 3 phenotypic measures of adiposity (body mass index (kg/m^2^), waist circumference (cm), and abdominal height (cm)), and significant association of another SNP within the VDR locus, the rs3782905, was observed. The mean waist circumference for women with the minor homozygote genotype of rs3782905 was 4.4 cm larger than for women with the common homozygous genotype [17]. In a Polish cohort of 351 postmenopausal women, no association of BsmI with BMI, total fat volume and visceral fat (as determined by total body dual-energy X-ray absorptiometry) was observed [18]. The observed differences in adiposity measures between our presented cohort and this closely geographically related Polish cohort can be explained by different methodology of measurement (X-ray absorptiometry in Polish study vs. bioimpedance in present study) or by different age structure of the cohorts, as the Polish cohort included only postmenopausal women, while our cohort included both men and women, and the premenopausal women were included, too.

Importantly, all of the studied polymorphisms were reported to have functional consequences. A cluster of three polymorphisms BsmI, ApaI, and TaqI at the 3′ end of the VDR gene are in near complete linkage disequilibrium among Caucasians. It is known that these polymorphisms do not influence protein sequence; however, there is indirect evidence that these polymorphisms could lead to altered transactivation [19]. Tajouri, et al. [20] and Partridge, et al. [21] investigated the functional effects of the FokI polymorphism, which has been demonstrated to alter the transcription initiation start site resulting in a shorter protein (F genotype), with demonstrated increased receptor activity. In a French cohort of adolescent girls, the EcoRV was associated with vitamin D status and calcium levels and it can be also suggested that due to its location in the promoter of the VDR gene, this SNP may have an important functional effect on expression of the gene [22]. In a novel study by Khan et al., the G allele of the rs4328262 in VDR region was associated with increased VAT volume (β = 45.7; *P* < 0.001), while the A allele of another SNP (rs11574070) was nominally associated with body fat percentage (β = 0.96; *P* = 0.002) in the African American population [23]. In this study, none of the VDR SNPs analyzed showed any link with WC or BMI, which is contradictory to our observations. As the study by Khan et al. investigated the African-American population, while our study looked on the white European population, it could be suggested that the effects of VDR on body composition could by co-dependent on the ethnicity of the subjects.

Hence, all of the mentioned polymorphisms have proved functional impact. In our study, we observed association of this SNP with all the three investigated adiposity phenotypes, i.e. waist circumference, sum of skin fold thickness and total % of body fat which contradicts the results of the study by Ochs-Balkom et al. [17]. However, the study by Osch-Balkom included only postmenopausal women recruited in frame of a case-control design for breast cancer, while the presented study included male and female population including premenopausal women.

## Strenghts and limitations of the study

The major strength of the study is the relatively large sample size, standardized phenotypic measurements performed by the trained specialists and 7-day food records obtained from the individuals. Also, our study included males as well as females and was not focused only on premenopausal females. On the other hand, the major limitation may be a monocentric character of study that included only white Europeans. Moreover, phenotypic data on circulating levels of 1,25 dihydroxyvitamin D and/or 25-hydroxivitamin D are not available from our study individuals and hence presumptions on possible influence of investigated VDR SNPs on vitamin D levels are only indirect and based on literature.

## Conclusion

In conclusion, data from this study confirm that the genetic variability in the VDR region may be an important factor influencing anthropometric parameters associated with obesity, i.e. waist circumference, sum of skin fold thickness and total % of body fat in the Central-European population.

## Abbreviations

KMO measurement: Kaiser-Meyer-Olkin measurement
VDR: Vitamin D Receptor
